# Outcome of Glomerular Disease Manifesting After Vaccination Against Severe Acute Respiratory Syndrome Coronavirus 2

**DOI:** 10.7759/cureus.63767

**Published:** 2024-07-03

**Authors:** Shuvam Roy, Anupma Kaul, Monika Yachha, Pallavi Prasad, Ravi S Kushwaha, Manas Patel, Narayan Prasad, Manoj Jain, Manas Ranjan Behera

**Affiliations:** 1 Department of Nephrology, All India Institute of Medical Sciences Raebareli, Raebareli, IND; 2 Department of Nephrology, Sanjay Gandhi Postgraduate Institute of Medical Sciences, Lucknow, IND; 3 Department of Pathology, Sanjay Gandhi Postgraduate Institute of Medical Sciences, Lucknow, IND

**Keywords:** post-vaccination glomerulonephritis, covaxin and covishield, post-vaccination glomerular disease, covid-19 vaccinations, sars-cov-2 (severe acute respiratory syndrome coronavirus -2), glomerulonephritis, glomerular disease

## Abstract

Introduction

Vaccination against severe acute respiratory syndrome coronavirus 2 (SARS-CoV-2) can upregulate the immune system and may contribute to glomerular disease (GD). Here, we describe a spectrum of GD that manifested following vaccination against SARS-CoV-2 (COVID-19 vaccinations).

Material and methods

This was a descriptive study of 10 cases enrolled between January 2021 and January 2023. Patients with biopsy-proven GD that manifested following COVID-19 vaccinations were included.

Results

We found 10 cases of biopsy-proven GD following the COVID-19 vaccination. This included five cases of minimal change disease (MCD), three cases of focal segmental glomerulosclerosis (FSGS), one case of C3 glomerulonephritis (C3GN), and one case of IgA nephropathy (IgAN). The pre-existing disease was found in the last two patients (IgAN and C3GN) who got unmasked following vaccination. We did not observe any relation between vaccine type (Covisheld; six cases vs. Covaxin; four cases) and GD. In most cases (8/10 cases, 80.0%), GD developed after a repeat dose (second or booster dose). The onset time following vaccination was typically less than a week, and even less following a repeat dose.

Conclusion

Post-vaccination GD can be either de novo or a flare-up of a pre-existing one. The onset time following vaccination was typically less than a week for both Covishield and Covaxin.

## Introduction

Coronavirus disease 2019 (COVID-19) is caused by infection with the severe acute respiratory syndrome coronavirus 2 (SARS-CoV-2). Different vaccines have been developed against SARS-CoV-2 and deployed in mass immunization campaigns worldwide. In India, Covishield (ChAdOx1 nCoV-19; manufactured by the Serum Institute of India) and Covaxin (BBV152; manufactured by Bharat Biotech) are two such vaccines that have been made available. The former is a replication-deficient adenovirus vaccine vector encoding the SARS-CoV-2 Spike (S) glycoprotein, while the latter is an inactivated whole virion vaccine [1,2].

There have been many case reports of the onset or relapse of glomerular disease (GD) occurring after the COVID-19 vaccination. This is attributed to the heightened off-target effects of the vaccine's immune response [3,4]. Most of them are following m-RNA vaccines. Here, we describe a spectrum of GD that manifested following non-mRNA COVID-19 vaccinations from a tertiary care hospital in India.

## Materials and methods

Patient selection and data collection

This descriptive study included 10 cases enrolled between January 2021 and January 2023. All patients who, for the first time, developed symptoms of GD following the COVID-19 vaccination and underwent a kidney biopsy were included in the study. Evidence of kidney disease before vaccination, as suggested by symptoms or investigation, a history of infection in the last three months before the current illness, or analgesic abuse were excluded from the study. The demographic and clinical information like age, gender, relevant past medical history, and laboratory findings like serum creatinine (mg/dL), 24-hour proteinuria (gm/day), urinalysis, and blood cell counts were recorded. Renal biopsy data, including immunofluorescence and electron microscopy, were also included. All patients were managed as per institute protocol. Details of immunosuppressants received by patients and responses to treatment were also noted. All patients were followed up until April 2024. Approval from the institute's ethics committee was obtained.

Definitions

Nephrotic syndrome was defined as nephrotic range proteinuria (in adults, proteinuria >3.5 g per 24 hours; in children, >40 mg/m2/hr) plus hypoalbuminemia and edema. Hematuria, hypertension, oliguria, and edema define nephritic syndrome. Complete remission (CR), partial remission (PR), relapse, steroid-resistant nephrotic syndrome (SRNS), frequent relapsing nephrotic syndrome (FRNS), and steroid-dependent nephrotic syndrome (SDNS) were defined as per the Kidney Disease Improving Global Outcomes (KDIGO) 2021 Clinical Practice Guidelines for the Management of Glomerular Diseases [5]. The index dose was the dose of COVID-19 vaccination that temporally correlates with the onset of GD, which can be either the first, the second, or the booster dose.

Statistical methods

This was a descriptive study of 10 patients. The data was presented in tabular form. Whenever required, frequency and percentage were used for categorical variables. Continuous variables were expressed as the median and range. Microsoft Excel Office 2021 (Microsoft Corporation, Redmond, Washington, United States) was used for statistical analysis.

## Results

Between January 2021 and January 2023, we came across 10 cases that developed glomerular illness after the COVID-19 vaccination. Demographic characteristics are represented in Table 1. Most of them were adults, except two. Six patients (60%) were male, while four (40%) were female. The median age was 32.5 years (range: 14 to 62 years). Four of them (40%) received Covaxin (BBV152), while six (60%) received Covishield. Two of them developed GD after the first dose, and the rest (80%) developed GD after repeat vaccination (seven cases after the second dose and one case after the booster dose). The onset of the first symptom of glomerular illness after vaccination was less than a week (range: one to six days). Fever was present in all cases except one. The nephritic syndrome was found in two cases (20%); one had gross hematuria. Nephrotic syndrome was found in eight cases (80%). Renal failure at presentation was found in three cases, and the rash was found in one case.

**Table 1:** Baseline characteristic F: female; M: male; HTN: hypertension; DM: diabetes mellitus; AKI: acute kidney injury; Mod: moderate; C3GN: C3 glomerulonephritis; FSGS: focal segmental glomerulosclerosis; IgAN: IgA nephropathy; MCD: minimal change disease; Mod IFTA: moderate interstitial fibrosis and tubular atrophy

Case	Age (Year)/ Sex	Co-morbidity	Vaccine	Index Dose	Interval Between First and Index Doses	Onset Time	Presenting Symptoms	Renal Syndrome	Kidney Biopsy	Follow Up Time (months)
1	39/F	HTN, Hypothyroid	Covishield	2nd	6 weeks	1 day	Fever, Rash, Edema	Nephritic Syndrome	C3GN with Mod IFTA	30
2	14/M	Nil	Covaxin	1st	-	4 days	Fever, Anasarca	Nephrotic Syndrome	FSGS	25
3	37/M	HTN	Covishield	Booster	26 weeks	3 days	Fever, Gross Hematuria	Nephritic Syndrome	IgAN with Mod IFTA	25
4	21/M	Nil	Covaxin	2nd	4 weeks	1 day	Fever, Anasarca	Nephrotic Syndrome	MCD	27
5	28/F	Nil	Covaxin	1st	-	6 days	Fever, Edema	Nephrotic Syndrome	MCD	28
6	55/M	HTN, Pulmonary Tuberculosis	Covishield	2nd	8 weeks	3 days	Edema	Nephrotic Syndrome with AKI	FSGS	20
7	39/F	HTN	Covishield	2nd	8 weeks	2 days	Fever, Edema	Nephrotic Syndrome	MCD	18
8	18/F	Nil	Covishield	2nd	6 weeks	2 days	Fever, Edema	Nephrotic Syndrome	MCD	24
9	62/M	DM, HTN	Covaxin	2nd	4 weeks	3 days	Fever, Edema	Nephrotic Syndrome	FSGS	22
10	22/M	Nil	Covishield	2nd	6 weeks	5 days	Fever, Edema	Nephrotic Syndrome	MCD	24

All cases underwent a renal biopsy. Minimal change disease (MCD) was the most common presentation (five cases; 50%). This was followed by focal segmental glomerulosclerosis (FSGS; three cases), C3 glomerulonephritis (C3GN; one case), and IgA nephropathy (IgAN; one case). The two patients with C3GN and IgAN, respectively, had mild to moderate interstitial fibrosis, suggesting that pre-existing disease went unmasked following vaccination. The median follow-up time was 24.5 months (18 to 30 months).

All patients received steroids as an initial treatment. Three out of five patients with MCD underwent CR, and two developed PR and FRNS, respectively, requiring the addition of tacrolimus. Two out of three patients with FSGS underwent PR with steroids, and CR was achieved with the addition of tacrolimus. One patient with FSGS with diabetes mellitus was found to have SRNS and achieved PR following the addition of mycophenolate mofetil. Mild renal failure was found in one case, with FSGS and recovered completely. The patient with IgAN and an acute tubular injury recovered completely with steroid-alone therapy. Mycophenolate mofetil was added to C3GN with renal dysfunction but responded partially concerning proteinuria and renal failure. All patients were optimized with renin-angiotensin aldosterone system (RAAS) blockers (ramipril or telmisartan). None of the patients developed progressive renal failure or end-stage renal disease (ESRD) until the last follow-up. Investigations and treatment are mentioned in Table 2.

**Table 2:** Investigation, treatment, and response to therapy CR: complete remission, PR: partial remission; FRNS: frequently relapsing nephrotic syndrome; SRNS: steroid resistant nephrotic syndrome; MMF: mycophenolate mofetil, Tac: tacrolimus

Case	Serum Creatinine (mg/dl)	Serum Albumin (gm/L)	Triglyceride/ Total Cholesterol (mg/dl)	Urine RBC (no/HPF)	24 Hours Urine Protein (gm)	Firstline Therapy	Response to 1^st^ Line Treatment	Second-line Therapy	Response to 2^nd^-line Therapy
1	1.9	3.21	149/237	18-20	6.0	Steroid	PR	MMF	PR
2	0.8	1.9	260/378	nil	5.6	Steroid	PR	Tac	CR
3	2.3	4.0	101/219	Plenty	3.5	Steroid	CR	-	-
4	1.1	2.1	334/402	nil	6.2	Steroid	FRNS	Tac	CR
5	0.7	2.0	432/328	nil	4.2	Steroid	CR	-	-
6	1.6	2.4	321/268	3-4	4.8	Steroid	PR	Tac	CR
7	1.0	1.7	572/348	nil	7.8	Steroid	PR	Tac	CR
8	0.6	2.8	228/356	nil	3.5	Steroid	CR	-	-
9	1.1	2.5	248/402	2-3	6.0	Steroid	SRNS	MMF	CR
10	0.8	2.4	378/503	nil	4.2	Steroid	CR	-	-

## Discussion

Vaccines, in general, have been known to be associated with a variety of types of GD. The same vaccine can lead to both proliferative and non-proliferative glomerular diseases, depending on the immune pathway activated by the vaccine. It is postulated that molecular mimicry between host peptides and antigenic targets leads to the formation of immune complex deposits [3]. This can lead to proliferative glomerulonephritis. Adjuvants in the vaccine can activate the inflammasome pathway, releasing cytokines like interleukin 1 and interleukin 18. This can up-regulate T helper cell types 1 and 17 and down-regulate T regulatory cells, potentially contributing to GD [4]. This can lead to non-proliferative glomerular disease. Vaccines against COVID-19 are also associated with GD, which can occur de novo or as a relapse of a preexisting disease.

In the current study, we found that ten patients had biopsy-proven GD following COVID-19 vaccination, including MCD (50%), FSGS (30%), C3GN (10%), and IgAN (10%). We did not observe any relation between vaccine type (Covishield; 60% vs. Covaxin; 40%) and GD. Notably, most cases (80%) developed after repeat vaccination (second or booster dose). Additionally, the onset time following vaccination was typically less than a week, even shorter after a repeat vaccination. These findings suggest a potential temporal relationship between vaccination and the development of GD. Around 60% of patients required the addition of a second immunosuppressant, and none of the patients had progressive renal failure until the last follow-up. Two patients with IgAN and C3GN had chronicity markers (mild to moderate interstitial fibrosis), suggesting vaccination unmasked the pre-existing GD.

Caza et al. published a series of 29 cases of GD occurring within one month of the first or second dose of the SARS-CoV-2 vaccination. Twenty-eight cases of de novo glomerulonephritis in the native kidney and one case of recurrent glomerulonephritis in the transplanted kidney were reported. The most familiar pathology was IgA nephropathy, followed by membranous nephropathy, crescentic glomerulonephritis, collapsing glomerulopathy, and MCD [6]. Canney et al. reported that there was a twofold higher risk of relapse of various GDs after the COVID-19 vaccination [7]. A systematic review by Wu et al. found that among post-COVID-19 vaccination GD, the most typical pathology was MCD, followed by IgAN and vasculitis [8].

Different vaccines have different mechanisms of action, but their target remains the spike protein of the SARS-CoV-2 virus. Glomerular disease has primarily been reported with mRNA-based vaccines, which are more immunogenic. Similar illnesses with other types of vaccines have been less frequently reported. Compared with conventional vaccines, mRNA vaccines induce greater activation of CD4+ T and CD8+ T cells and higher production of cytokines [9]. Moreover, mRNA vaccines may stimulate innate and adaptive immunity more than inactivated vaccines [10]. Here, we describe GD occurring in close temporal relation to Covishield and Covaxin, which may have different mechanisms of action.

Reviewing the literature, we find that the case series by Caza et al. included one patient with MCD occurring after vaccination with the ChAdOx1 nCoV-19 vaccine, which is marketed in India as Covishield [6]. Krishna et al. reported a case of MCD after ten days of the first dose of Covishield [11]. Biradar et al. described a similar case of post-Covishield MCD [12]. Fenoglio et al. reported two instances of IgAN and one case of vasculitis after administering the ChAdOx1 nCoV-19 vaccine [13]. Reports of GD after Covaxin administration are even rarer. Mahajan et al. described a collapsing FSGS occurring ten days after the first dose of Covaxin administration [14]. Prema et al. reported two cases of glomerulonephritis positive for both the anti-neutrophil cytoplasmic antibody (ANCA) and the anti-glomerular basement membrane (anti-GBM) antibody, which occurred within two weeks after administration of the first and second doses of Covaxin, respectively [15].

Temporal correlation does not imply causation. Indeed, the presence of features of chronicity in kidney biopsy points to the existence of previously undiagnosed renal pathology. However, even in such a case, vaccination may lead to the disease's flaring and unmasking due to its effects on the immune system, as we observed in two cases with IgAN and C3GN, respectively. As stated earlier, this has been corroborated by clinical research, which showed an increased risk or relapse of up to five percent for different GD post-vaccination [7].

However, the study has its limitations. With a tiny sample size and a lack of a control population, it isn't easy to establish the statistical significance of the above observations. Collaboration with other centers in India and abroad could have increased the power of our study.

## Conclusions

Post-COVID-19 vaccination-associated glomerular disease can have varied pathologies. Both proliferative and non-proliferative glomerulonephritis can occur after vaccination with Covishield or Covaxin. Glomerular diseases can be either de novo or flare-ups of pre-existing illnesses. The onset time following vaccination was typically less than a week for both Covishield and Covaxin. Patients often respond to standard medications for these conditions. Despite the small risk of such diseases, vaccination is highly recommended for the general population, given the enormous benefits.
